# Lipid binding promotes the open conformation and tumor-suppressive activity of neurofibromin 2

**DOI:** 10.1038/s41467-018-03648-4

**Published:** 2018-04-06

**Authors:** Krishna Chinthalapudi, Vinay Mandati, Jie Zheng, Andrew J. Sharff, Gerard Bricogne, Patrick R. Griffin, Joseph Kissil, Tina Izard

**Affiliations:** 1 Department of Integrative Structural and Computational Biology, The Scripps Research Institute, Jupiter, FL 33458 USA; 2 Department of Molecular Medicine, The Scripps Research Institute, Jupiter, FL 33458 USA; 3 Global Phasing Ltd., Sheraton House, Castle Park, Cambridge, CB3 0AX United Kingdom

## Abstract

Neurofibromatosis type 2 (NF2) is a tumor-forming disease of the nervous system caused by deletion or by loss-of-function mutations in *NF2*, encoding the tumor suppressing protein neurofibromin 2 (also known as schwannomin or merlin). Neurofibromin 2 is a member of the ezrin, radixin, moesin (ERM) family of proteins regulating the cytoskeleton and cell signaling. The correlation of the tumor-suppressive function and conformation (open or closed) of neurofibromin 2 has been subject to much speculation, often based on extrapolation from other ERM proteins, and controversy. Here we show that lipid binding results in the open conformation of neurofibromin 2 and that lipid binding is necessary for inhibiting cell proliferation. Collectively, our results provide a mechanism in which the open conformation is unambiguously correlated with lipid binding and localization to the membrane, which are critical for the tumor-suppressive function of neurofibromin 2, thus finally reconciling the long-standing conformation and function debate.

## Abstract (web summary)

Neurofibromin 2 (NF2) is a tumour suppressor that inhibits cell growth. Here the authors combine functional, biochemical, and structural studies and show that lipid-bound NF2 adopts an open conformation and that NF2 lipid binding is required for inhibition of cell proliferation.

## Introduction

Mutations in the *NF2* gene cause Neurofibromatosis type 2 (NF2), a nervous system tumor-forming disease that is characterized by the development of bilateral vestibular schwannomas^1^. NF2 has an incidence of 0.004% of the population, where it is dominantly inherited. However, more than half of NF2 patients develop the disease from loss-of function due to de novo mutations^1,2^. NF2 manifests itself in the development of multiple schwannomas on cranial and peripheral nerves as well as of ependymomas and meningiomas that can only be treated surgically where loss-of function of the involved nerve is often an unpreventable consequence. Thus, chemotherapies to slow or eliminate tumor formation are urgently needed, especially since NF2 mutations that result in inactivation of its gene product, neurofibromin 2 (also known as schwannomin or merlin for moesin-ezrin-radixin-like protein), are also found in spontaneous meningiomas and schwannomas and several other types of cancers, such as breast, colorectal, clear cell renal cell carcinoma, hepatic, glioma multiforme, mesothelioma, and prostate. Thus, understanding the neurofibromin 2 activation mechanism will increase our knowledge of the role of NF2 mutations in cancer and might eventually aid prognosis and future chemotherapeutic therapies.

**Fig. 1:**
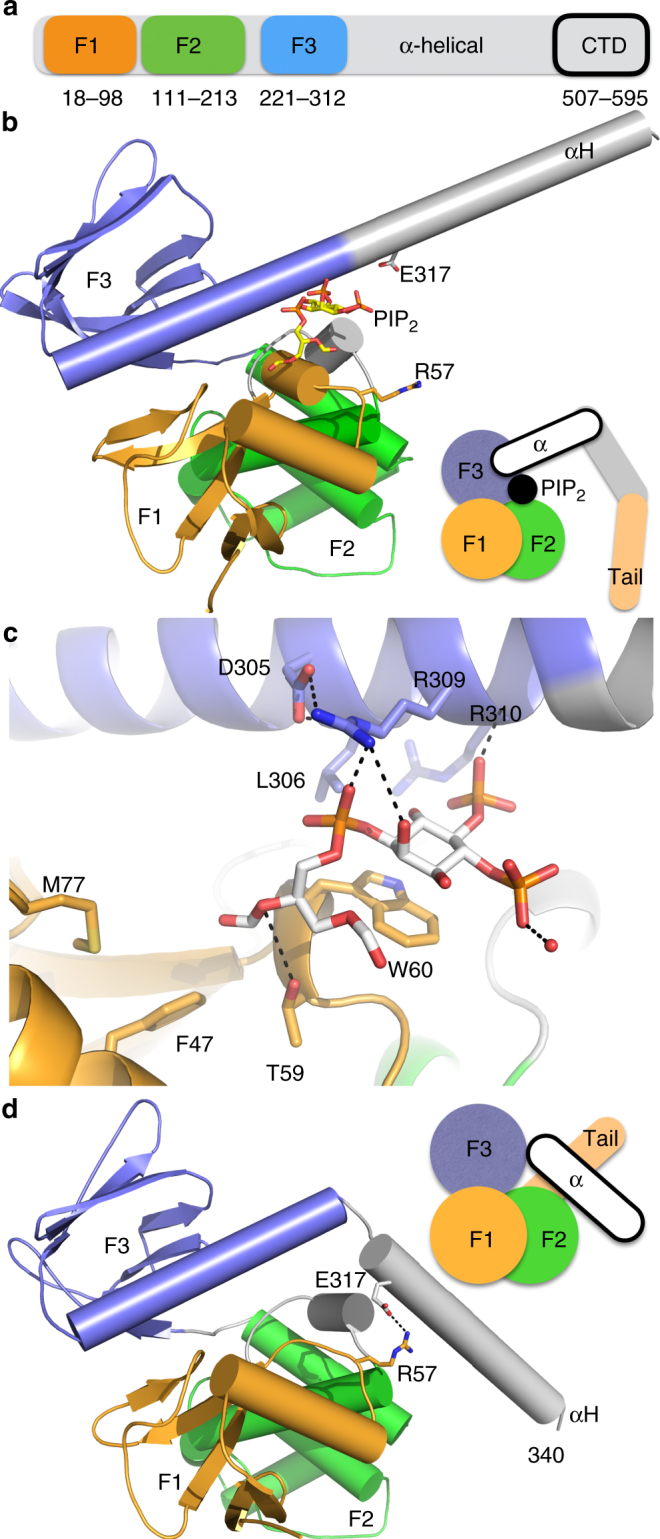
Lipid binding to neurofibromin 2 causes major conformational changes. **a** Schematic depiction of the domain organization of full-length neurofibromin 2. The F1, F2, and F3 FERM subdomains and the *C*-terminal domain (CTD) are indicated that are connected by the central α-helical domain. **b** The 2.61 Å PIP_2_-bound neurofibromin 2 structure. The F1 subdomain (residues 18–98) is shown in light orange, F2 (residues 111–213) in green, and F3 (residues 221–312) in blue. The α-helix from the central helical domain, αH, is shown in gray (residues 315–339). The cartoon illustrates binding of PIP_2_ to full-length neurofibromin 2 with its head–tail interaction severed. **c** Close-up view of the PIP_2_ binding site. The carbon atoms of PIP_2_ are shown in gray, of the neurofibromin 2 F1 subdomain in orange, and F3 in blue. Hydrogen bonds are indicated and key binding residues are labeled. **d** View of the closed FERM neurofibromin 2 structure (PDB entry 1isn^34^) in the same orientation as our lipid-bound structure shown in panel **b**. The electrostatic interaction between E317 OE2 and R57 NH2 is indicated (and the distance is 2.9 Å) that is severed in our lipid-bound structure shown in **b**. The cartoon highlights the distinct conformation of α-helix αH from the central domain

Neurofibromin 2 belongs to the FERM (band 4.1, ezrin, radixin, moesin) gene family characterized by an *N*-terminal FERM domain (Fig. 1a) that binds to the plasma membrane, a central α-helical domain, and a *C*-terminal tail domain that binds to the cortical actin cytoskeleton^3^. In contrast, neurofibromin 2 has a unique cytoskeleton binding site on its *N*-terminal domains^4^. By directly or indirectly linking the actin cytoskeleton, neurofibromin 2 is proposed to be involved in organization and maintenance of cytoskeleton architecture beneath plasma membrane. Neurofibromin 2 is a unique tumor suppressor protein that inhibits cell growth both from plasma membrane and in the nucleus upon cell confluency^5,6^. For example, research carried out by several groups indicated that neurofibromin 2 suppresses cellular growth either inhibiting conversion of Ras/Rac-GDP bound forms to GTP bound form or by hampering Ras recruitment at the plasma membrane^7–9^. Neurofibromin 2 also attenuates growth factor receptors expression and activity at the plasma membrane in both drosophila and mammals^10^ and exerts its growth-suppressive function in the nucleus, where it inhibits the DCAF ubiquitin ligase activity^11^. Neurofibromin 2 is a major regulator of Hippo signaling that is involved in the conserved kinase cascade that inhibits organ overgrowth through cytoplasmic of sequestration of YAP by phosphorylation, and blocking its ability to promote transcriptional enhancer activation domain (TEAD)–dependent transcription of genes involved in proliferation and survival such as *CTGF*, *Cyr61*, *Axl*, *BIRC5*, and *PTGS2*^12–14^.

The neurofibromin 2 head–tail interaction is critical for controlling the tumor-suppressive function of neurofibromin 2^15,16^, which seems also to be regulated by phosphorylation. Specifically, protein kinase A and p21-activated kinases phosphorylate S518 residing on the tail domain, thereby inactivating the tumor-suppressive activity of neurofibromin 2^17–20^, while the myosin phosphatase MYPT1-PP1δ activates its tumor suppressor function by dephosphorylating S518^21^. However, the relationship between neurofibromin 2 activity and conformation is highly controversial^6^. Originally, by extrapolation from other ERM proteins, neurofibromin 2 was assumed to be phosphorylated at the plasma membrane in its open conformation to promote receptor mediated signaling events that control cell proliferation and survival^22,23^. However, in its closed conformation, neurofibromin 2 does not seem to bind to growth factor receptors in its supposedly closed, tumor suppressor active, phosphorylated form^24,25^. More recently, fluorescence resonance energy transfer, mutagenesis, and small-angle neutron scattering studies suggested that unphosphorylated neurofibromin 2 is in its closed conformation that can interact with binding partners including phosphatidylinositol 4,5-bisphosphate (PIP_2_)^26–28^.

Here we report the crystal structure of the neurofibromin 2 *N*-terminal domain in complex with PIP_2_, which reveals a large conformational change upon lipid binding. Our hydrogen-deuterium exchange experiments with full-length neurofibromin 2 confirm that lipid binding severs the neurofibromin 2 head–tail interaction. Finally, by introducing our structure-based and patient-derived mutagenesis of neurofibromin 2 in live cells, we find that the neurofibromin 2 conformational changes associated with lipid binding and potentially membrane attachment are necessary for neurofibromin 2 inhibition of cell proliferation and that lipid binding and membrane attachment are necessary for neurofibromin 2 to exert its cell growth inhibiting functions and to inhibit YAP activity.

## Results

### Lipid binding alters the conformation of neurofibromin 2

Neurofibromin 2 localizes to membrane rafts^29,30^, the hotspot for many signaling events, and its membrane attachment has been shown to be important for its anti-proliferative functions^31–33^. To gain detailed molecular insight into this important interaction, we determined the 2.71 Å crystal structure of neurofibromin 2 (residues 1–339) in complex with PIP_2_ (Fig. 1b**;** Supplementary Tables 1, 2). The crystal structure contains two polypeptide chains, two polyethylene glycols, two phosphates, and two PIP_2_ molecules in the asymmetric unit (Supplementary Fig. 1, 2). The two polypeptide chains in the asymmetric unit are very similar and can be superimposed with *r.m.s.d.* of 0.219 Å for 2,309 atoms (Supplementary Fig. 3a). In both polypeptide chains, the PIP_2_ inositol head group is sandwiched between Trp-60 and Arg-309, the 5′ inositol phosphate interacts with the backbone of Arg-310 and the 1-phosphate group is in hydrogen-bonding distance to Arg-309 (Fig. 1c, d**;** Supplementary Fig. 3b**;** Supplementary Table 3**)**. The oxygen atoms of the diacylglycerol interact with the hydroxyl group of Thr-59.

Akin to the known apo structure (PDB entry 1isn^34^), our PIP_2_-bound structure comprises the entire FERM domain and the following additional α-helix from the helical domain (αH; residues 315–340) (Fig. 1d). While the lipid-bound FERM domain part of the structure (residues 21–312) resembles the apo FERM structure, the α-helix of the helical domain αH undergoes a large structural change and adopts a distinct conformation. In the apo structure, a short loop (residues 312–315) allows αH to interact with the F1 FERM subdomain (specifically by electrostatic interactions of Arg-57 with Asp-314). Upon PIP_2_ binding, the acyl group stacks with the side chain of Arg-57 at this interface and severs the FERM and αH interaction further by exposing its 5′ phosphate group into the solvent. This causes the loop (residues 312–315) that keeps αH interacting with F1 in the apo structure, to become helical, and thereby extending the last F3 α-helix (residues 290–312) to form one continuous long α-helix (residues 290–339) instead in the lipid-bound structure by a ~60° relative rotation.

### Lipid binding severs the neurofibromin 2 head–tail interaction

**Fig. 2:**
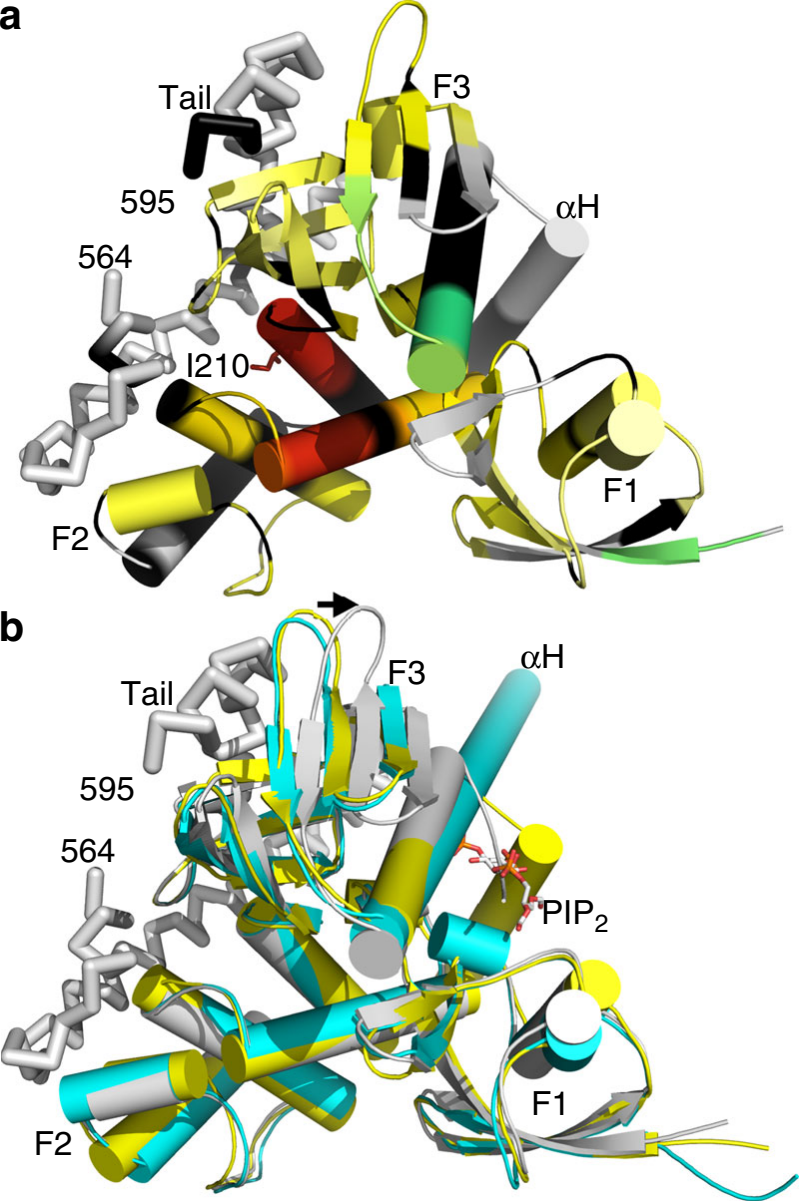
Hydrogen-deuterium exchange (HDX) and mass spectrometry mapping onto the neurofibromin 2 head/tail structure (PDB entry 4zrj^35^). **a** Differential deuterium exchange (ΔHDX) between apo and PIP_2_ bound residues are color coded from red to blue with warm colors representing increased conformational dynamics (red being the relative highest D_2_O uptake) and cool colors representing decreased conformational dynamics (blue being the lowest D_2_O uptake); gray, no statistically significant changes between compared conditions; black, regions that have no sequence coverage or include prolines that have no amide hydrogen exchange activity. I210 that binds the tail domain and shows largest degree of deprotection upon binding to PIP_2_ is shown in sticks and labeled. The tail domain is shown as a Cα-trace. **b** Superposition of the apo structure (yellow) onto the head–tail (PDB entry 4zrj^35^; gray), and PIP_2_-bound structures (cyan). PIP_2_ is shown as spheres and the tail domain as a Cα-trace. The arrow indicates the movement upon binding of the tail domain of 7.3 Å at the tip of loop (residue K279)

To confirm our structural findings in solution, we performed hydrogen-deuterium exchange (HDX) and mass spectrometry experiments on the full-length protein in the presence and absence of PIP_2_ micelles (Supplementary Fig. 4, Supplementary Data 1). In agreement with our neurofibromin 2/PIP_2_ and the head/tail crystal structures, the regions that showed most differential deuterium exchange or the highest deprotection profiles in the full-length apo versus PIP_2_-bound proteins were residues 207–213, where particularly Ile-210 was identified in engaging in hydrophobic interactions with the tail domain^35^ (Fig. 2a). A further region that showed the most deprotection (residues 122–126) is not involved in the head–tail interface which thus likely presents an interaction interface with the central helical domain that is missing in the crystal structures. Thus, PIP_2_ releases the tail domain as well as parts of the central helical domain.

Superposition of the apo (PDB entry 1isn^34^) and the FERM/tail structures (PDB entry 4zrj^35^) onto our lipid-bound structure further confirms that our lipid-bound structure is in its open conformer (Fig. 2b). While all three structures are similar, the F3 subdomain is moved over 7 Å in the head/tail structure compared to the lipid-bound and apo structures to allow binding of the tail domain. Importantly, in our lipid-bound structure, the F3 subdomain is in the conformer seen in the apo structure in the space that is occupied by the tail domain in the head/tail structure. Thus, lipid binding is consistent with the unbound, open conformation.

### The neurofibromin 2/PIP_2_ open conformation is the active state

**Fig. 3:**
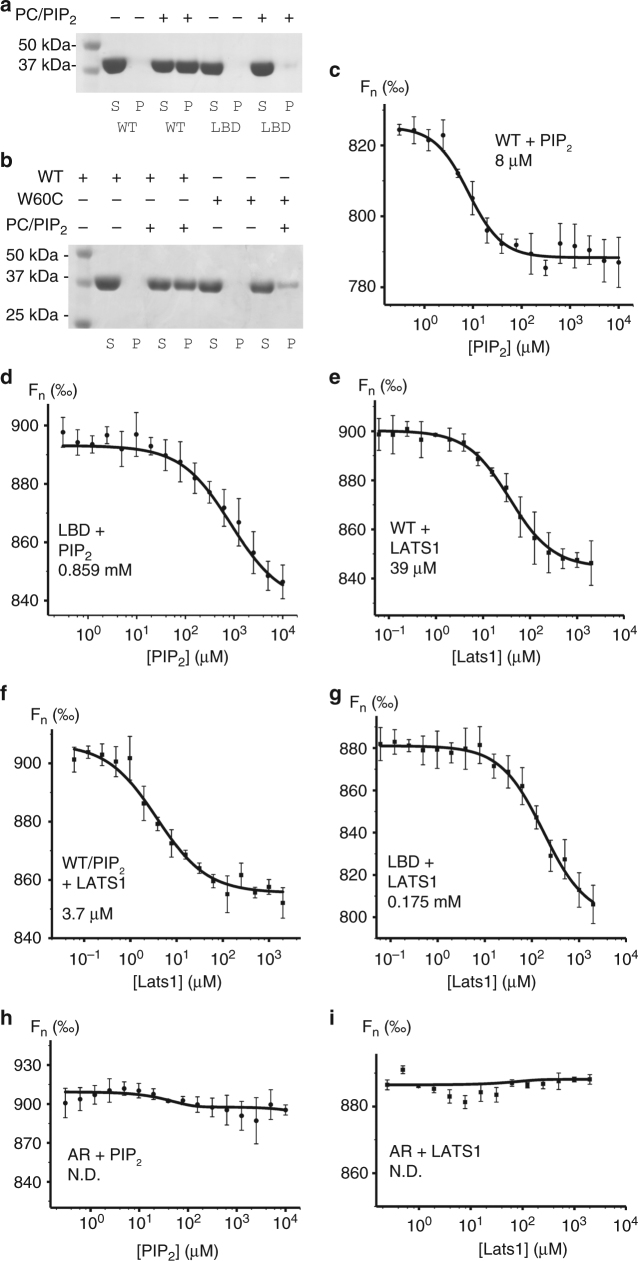
The conformation of neurofibromin 2 dictates its binding. **a** Lipid co-sedimentation analysis of the PIP_2_ binding to wild type and our lipid binding deficient mutant neurofibromin 2. wild-type neurofibromin 2 (residues 1–339) is soluble in the absence of PIP_2_ and pellets in the presence of PIP_2_. Mutant (T59V, W60E, R309Q, R310Q) neurofibromin 2 (residues 1–339) remains soluble in the absence and presence of PIP_2_. S supernatant, P pellet, WT wild type, LBD lipid binding deficient. **b** Lipid co-sedimentation analysis of the PIP_2_ binding to wild-type and disease-derived mutant neurofibromin 2. Wild-type neurofibromin 2 (residues 1–339) is soluble in the absence of PIP_2_ and pellets in the presence of PIP_2_. Mutant (W60C) neurofibromin 2 (residues 1–339) is soluble in the absence of PIP_2_, while a small fraction pellets in the presence of PIP_2_. S supernatant, P pellet, WT wild type. Microscale thermophoresis (MST) measurements show the binding of PIP_2_ to **c** wild-type full-length neurofibromin 2 (*K*_d_ = 8.02 ± 0.91 μM) or to **d** our lipid binding deficient (LBD) mutant (T59V, W60E, R309Q, R310Q; *K*_d_ = 859.23 ± 184.65 μM). MST measurements show binding of LATS1 (residues 69–100) to **e** wild type (*K*_d_ = 39.31 ± 4.25 μM), **f** the neurofibromin/PIP_2_ complex (*K*_d_ = 3.77 ± 0.72 μM), or **g** to our LBD neurofibromin 2 mutant (*K*_d_ = 175.54 ± 34.49 μM). No binding was observed for the artificially closed A585W-R588K (AR) mutants to **h** PIP_2_, or **i** LATS1. Error bars represent ±S.D., *n* = 3 (three independent measurements with the same laser power)

Isothermal calorimetry data for the FERM domain and full-length proteins with PIP_2_ micelles determined binding constants of 3.3 μM and 0.65 μM, respectively^28^. To determine the correlation between the open lipid-bound conformation and tumor-suppressive function, we first generated a lipid binding deficient mutant (T59V, W60E, R309Q, R310Q) neurofibromin 2 (Fig. 3a, Supplementary Fig. 5). By lipid co-sedimentation assay, we found that wild-type neurofibromin 2 binds PIP_2_, while our lipid binding deficient mutant did not. Thus, residues Thr-59, Trp-60, Arg-309, and Arg310 are crucial for neurofibromin 2 attachment to the cell membrane.

We next sought to understand the effect of the patient-derived W60C neurofibromin 2 mutant onto binding to the plasma membrane given that a key lipid binding residue, Trp-60, was replaced. We found that the W60C mutant bound lipids about four times weaker compared to the wild type as judged by our co-sedimentation assay and gel electrophoresis (Fig. 3b, Supplementary Fig. 5). Unfortunately, we were unable to obtain soluble proteins for other disease relevant mutations near the lipid binding pocket (F62S and L64P) to evaluate their effects on membrane attachment.

To link the conformational effects of lipid binding on neurofibromin 2 to its function, we determined the binding of full-length GST-tagged wild type and mutant neurofibromin 2 to the Hippo pathway kinase LATS1 and PIP_2_ by microscale thermophoresis (MST). Wild-type neurofibromin 2 bound PIP_2_ with 8 μM (Fig. 3c), while we obtained a weak affinity (*K*_d_ of 0.859 mM) for our lipid binding deficient mutant with respect to PIP_2_ (Fig. 3d). The LATS1 kinase bound wild-type neurofibromin 2 with 39 μM (Fig. 3e) and this interaction was increased about ten-fold in the presence of PIP_2_ (Fig. 3f). Our lipid binding deficient mutant bound LATS1 about 4.5 times weaker (0.175 mM; Fig. 3g) compared to wild-type neurofibromin 2. Consistent with the notion that lipid binding induces the open conformer, the constitutively closed neurofibromin 2 A585W/R588K mutant did not bind to PIP_2_ (Fig. 3h) or LATS1 (Fig. 3i). Thus, PIP_2_ binding releases the neurofibromin 2 tail domain from its head domain to then allow the open neurofibromin 2 conformer to recruit the downstream interaction partners in the Hippo signaling.

**Fig. 4:**
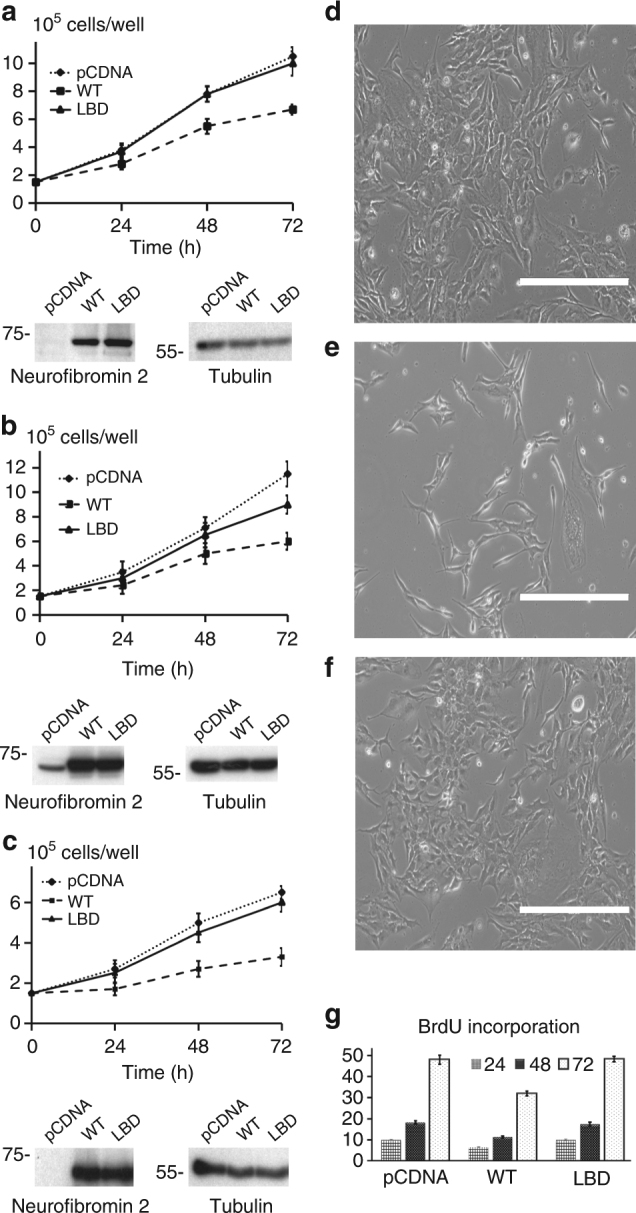
Lipid binding deficient mutants of neurofibromin 2 display impaired inhibition of cell proliferation. **a** SC4, **b** HEK293T, or **c** hSCλ-shNF2 cells were transfected with expression vectors for wild type and lipid binding deficient neurofibromin 2 or empty vector control (pCDNA). Total cell numbers were counted over 72 h. Means of each data point were calculated from three independent biological replicates conducted in triplicate. Error bars represent ± S.D. Immunoblot analysis was used to verify similar expression levels of the indicated neurofibromin 2 alleles. Tubulin was used as a control. The blots shown are representative of three biological replicates. For SC4 cells: difference between pCDNA and lipid binding deficient neurofibromin 2, < 0.7680 (i.e., not significant); pCDNA and wild-type neurofibromin 2, < 0.0001 (i.e., significant); lipid binding deficient and wild-type neurofibromin 2 proteins, < 0.0001 (i.e., significant). For HEK293T cells: difference between pCDNA and lipid binding deficient neurofibromin 2, <0.0013 (i.e., significant); pCDNA and wild-type neurofibromin 2, <0.0001 (i.e., significant); lipid binding deficient and wild-type neurofibromin 2 proteins, <0.0001 (i.e., significant). For hSCλ-shNF2 cells: difference between pCDNA and lipid binding deficient neurofibromin 2, <0.2476 (i.e., not significant); pCDNA and wild-type neurofibromin 2, <0.0001 (i.e., significant); the lipid binding deficient and wild-type neurofibromin 2 proteins, <0.0001 (i.e., significant). Scalebar size is 400 μm. **d**–**f** Phase contrast microscopy images, taken at the 72 h time point, of SC4 cells that were used in the BrdU cell proliferation assay. SC4 cells transfected with **d** pCDNA, **e** neurofibromin 2, and **f** the neurofibromin 2 lipid binding deficient mutant. **g** Cells expressing lipid binding deficient mutants of neurofibromin 2 display impaired inhibition of BrdU incorporation. SC4 cells were transfected with expression vectors for wild type and lipid binding deficient neurofibromin 2 or empty vector control (pCDNA) and BrdU incorporation was assessed over 72 h. Means of each data point were calculated from three independent biological replicates conducted in triplicate. Error bars represent ± S.D. Difference between pCDNA and our lipid binding deficient mutant neurofibromin 2, <1.0000 (i.e., not significant); pCDNA and wild-type neurofibromin 2, <0.000 (i.e., significant); wild type and our lipid binding deficient mutant, <0.0001 (i.e., significant)

To assess the functional significance of lipid binding to neurofibromin 2, we examined the ability of our lipid binding deficient neurofibromin 2 to inhibit cell growth and compared this to neurofibromin 2 wild type (Fig. 4a–f, Supplementary Fig. 6). *NF2*-null mouse schwannoma cells (SC4) or immortalized human Schwann cells that stably suppress neurofibromin 2 expression (hSCλ-shNF2), or immortalized human embryonic kidney cells (293 T), were transfected with expression plasmids for wild type and mutant neurofibromin 2 and cell numbers were counted up to 72 h. While expression of wild-type neurofibromin 2 suppressed the growth of these cells, the lipid binding deficient neurofibromin 2 expressing cells grew at rates similar to controls.

To establish whether cells expressing the lipid binding deficient mutant neurofibromin 2 affect cell proliferation rates compared to wild-type neurofibromin 2 transfected cells, BrdU incorporation was assessed over a 72-hour period (Fig. 4g). In agreement with the cell counting experiments, wild-type neurofibromin 2 cells displayed reduced rates of BrdU incorporation compared to cells transfected with expression plasmids for the control and the lipid binding deficient mutant. This suggests that conformational changes associated with lipid binding and potentially membrane attachment are necessary for neurofibromin 2 inhibition of cell proliferation.

**Fig. 5:**
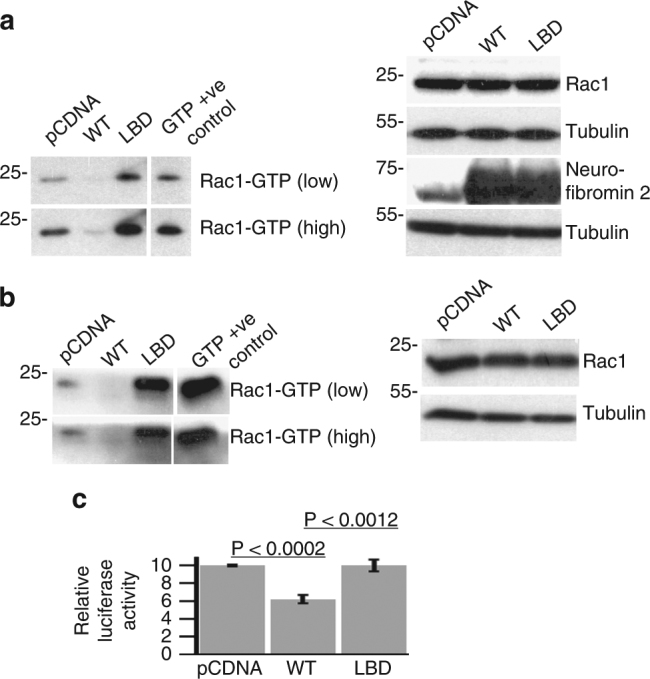
The lipid binding deficient mutant of neurofibromin 2 displays impaired inhibition of Rac1 activation and YAP activity. **a** 293 T or **b** SC4 cells were transfected with expression vectors for wild type or lipid binding deficient neurofibromin 2 or empty vector control (pCDNA) and levels of active Rac1 (Rac1-GTP) were assessed after 48 h. Levels of total Rac1, neurofibromin 2, and tubulin were assessed as controls. The blots shown are representative of three biological replicates. **c** HEK293T cells were transfected with expression vectors for wild type or lipid binding deficient neurofibromin 2 or empty vector control (pCDNA) along with YAP-driven luciferase and Renilla luciferase reporters. Activity of the luciferase reporter was assessed 24 h post transfection. Means of each data point were calculated from three independent biological replicates conducted in triplicate. Error bars represent ± S.D

We have previously shown that neurofibromin 2 can function as a regulator of small G-proteins from the Rac1/cdc42 family and of the Hippo-YAP pathway^9,36^. Thus, we first assessed whether expression of the lipid binding deficient mutant can reduce levels of active Rac1 (Rac1-GTP) and compared this to wild-type neurofibromin 2 (Fig. 5a, b Supplementary Figure 7). As expected, transfection of either SC4 or 293 T cells with an expression vector for wild-type neurofibromin 2 resulted in decreased Rac1-GTP levels. However, expression of the neurofibromin 2 lipid binding deficient mutant did not significantly alter the levels of Rac1-GTP in transfected cells. Thus, lipid binding and membrane attachment are necessary for neurofibromin 2 to exert its cell growth inhibiting functions.

As mentioned above, neurofibromin 2 is a key component of Hippo signaling and through which it regulates YAP transcriptional activity by inhibiting its translocation into the nucleus^14^. To assess the impact of the lipid binding deficient mutants against YAP activity, we co-transfected the neurofibromin 2 expression plasmids along a luciferase reporter plasmid for YAP-TEAD transcriptional activity, which served as a reporter for YAP activity (Fig. 5c). As expected, the expression of wild-type neurofibromin 2 resulted in a reduction of the YAP-TEAD reporter activity compared to the control. However, expression of the lipid binding mutant of neurofibromin 2 did not inhibit reporter activity. Thus, as in the case of active Rac1, lipid binding and membrane attachment are necessary for neurofibromin 2 to inhibit YAP activity.

## Discussion

Stabilization of neurofibromin 2 at the membrane is an important process that regulates several cellular functions of neurofibromin 2. For example, the direct neurofibromin 2-paxillin interaction has been shown to be involved in the stabilization of neurofibromin 2 at the plasma membrane, which is important for its tumor-suppressive functions^37^. Despite the mapping of the paxillin-binding site on neurofibromin 2 to residue W60 by biochemical and cell biology studies, it is unclear how the stabilization occurs at the cell membrane^38^. Significantly, a mutation of this tryptophan residue to a cysteine was found to be present in NF2 patients^38^. Based on our neurofibromin 2/PIP2complex structure, residue W60 is in fact involved in binding to the lipid, and thereby stabilizing the adhesion complex by anchoring neurofibromin 2 to the plasma membrane. Indeed, the NF2 patient derived W60C mutant binds lipids about four-fold weaker compared to wild type, suggesting the importance of W60 in binding to the plasma membrane. The sequestration to the cell membrane might aid paxillin recruitment to cell adhesion sites where it binds directly to neurofibromin 2 and stabilizes the adhesion complex.

The structural model of auto-inhibition and cycling between closed/resting and open/active conformational states of ERM proteins is often employed to explain the function of neurofibromin 2^39,40^. While the open conformation of ERM proteins represents their active states, the evidence that this is also the case for neurofibromin 2 is debated^41,42^. Previous studies in mammalian cells have shown that S518 is phosphorylated and that the S518D phosphomimic mutation blocks the intermolecular FERM-tail interactions^17,43^. However, S518 is located far from the head–tail domain interface in the closed A585W, R588K (“AR”) neurofibromin 2 mutant structure^27,28,35^. Studies in Drosophila indicate that deletion of the tail domain results in a constitutively active neurofibromin 2 that provides full genetic rescue on a null background^44^ suggesting that, like ERM proteins, the open form of neurofibromin 2 is active in vivo^27^.

**Fig. 6:**
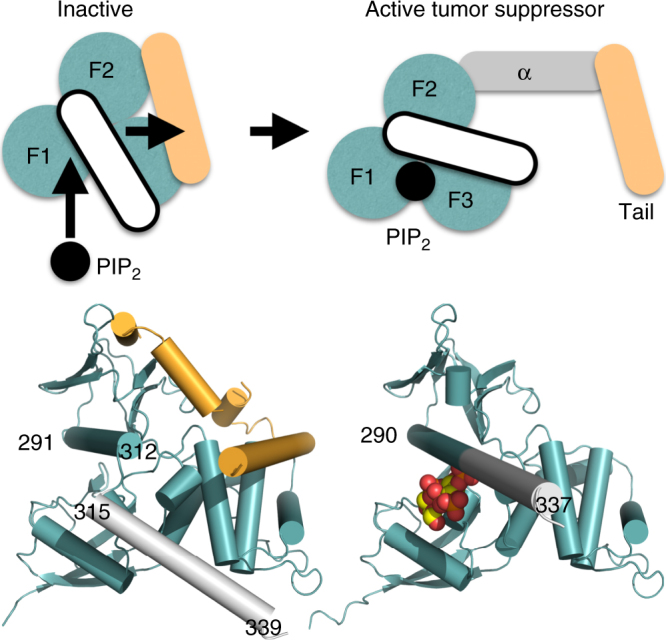
Activation of the neurofibromin 2 tumor-suppressive function. In its inactive state, neurofibromin 2 is in a closed conformation through interactions of the FERM domain (teal; F1, residues 18–98; F2, residues 111–213; F3, residues 220–312) and the tail domain (pale orange). The α-helix *C*-terminal of F3 (residues 315–339; white) does not interact with the tail domain. PIP_2_ binds to F1 and the last α-helix (residues 291–312; not depicted) of F3, thereby causing the last F3 α-helix (residues 291–312) and the following α-helix αH (residues 315–339) to rearrange as one long and continuous α-helix (residues 290–337), thereby displacing the tail domain and severing the head–tail interaction which results in active tumor suppressor functions. The central α-helical domain is shown in gray. The head/tail neurofibromin 2 crystal structure (head structure from PDB entry 1isn^34^; tail structure from PDB entry 4zrj^35^) is shown below the schematic on the bottom left and our PIP_2_-bound structure on the bottom right

Our crystal structure of neurofibromin 2 in complex with PIP_2_ now solve this long-standing debate (Fig. 6) as it reveals the lipid induced severing of the head–tail interaction which is supported by hydrogen-deuterium exchange data of full-length neurofibromin 2. Upon binding, PIP_2_ rearranges a loop into an α-helix, releases the interaction of the first α-helix αH of the central domain with the FERM domain to instead form one continuous long α-helix that ultimately displaces the tail domain. Our data uncover a mechanism of conformational changes that transition the closed form of neurofibromin 2 to its open conformer and link its dynamic states to previously characterized neurofibromin 2 functions. For example, it was shown previously that binding of Angiomotin to the neurofibromin 2 *C*-terminal region (residues 451–550) releases the auto-inhibition of the head/tail interaction and exposes the neurofibromin 2 FERM domain to interact with LATS1/2^35^. Although conceptually similar, our data indicate a model where binding of PIP_2_ to the FERM domain itself releases an auto-inhibitory *C*-terminal domain lock and might allow neurofibromin 2 to interact with its interacting partners such as LATS1/2 and Angiomotin. A systematic screen for identification of interacting partners of wild-type neurofibromin 2 versus our lipid binding deficient mutant (T59V, W60E, R309Q, R310Q) might reveal additional mechanistic insights into neurofibromin 2 tumor suppressor functions.

Our HDX data show that both in the absence and presence of lipid, the neurofibromin 2 tail domain exchanges deuterium, which is consistent with the weak head–tail interaction (*K*_d_ of 3 μM) compared to the other ERM proteins where for example the binding affinity of the moesin tail domain for its FERM domain is 16 nM^35^. Thus, in its unbound state, neurofibromin 2 is in a semi-open conformation which is consistent with structural and biochemical studies^16,27,35^. Our HDX data clearly show that upon addition of PIP_2_, the neurofibromin 2 FERM domain is fully exposed with moderate to high deuterium exchanges of FERM domain residues whereby the differential HDX (HDX_apo_−HDX_PIP2_) analysis shows that the upon addition of lipid micelles, the neurofibromin 2 FERM and tail domains are completely severed. This observation is in agreement with the low resolution SANS experiments^28^. In contrast to wild-type neurofibromin 2, our binding data show that the constitutively closed AR mutant does not bind lipids or LATS1 suggesting that neurofibromin 2 is not fully closed as also suggested by our HDX data. Upon binding to PIP_2_, neurofibromin 2 binds LATS1 ten-fold tighter, implying that PIP_2_ fully releases the neurofibromin 2 head–tail interaction. Collectively, in its unbound state, neurofibromin 2 exists in a semi-open conformation and phosphoinositides induce the open conformation.

Our functional studies suggest that the lipid binding deficient mutations significantly impair the functions of neurofibromin 2, as these mutations impaired the ability of neurofibromin 2 to inhibit cell proliferation. Neurofibromin 2 has been shown to mediate anti-proliferative effects through several downstream signaling pathways. These include signaling through small G-proteins at regions of cell-cell contacts. Neurofibromin 2 and Rich1 bind to Angiomotin at junctional structures and inhibit Rac1 and downstream signaling into the MAPK pathway^9^. In addition, neurofibromin 2 has been shown to regulate signaling through YAP on multiple levels^13,36,45^. Our findings demonstrate that both lipid binding deficient and the open forms of neurofibromin 2 are impaired in their ability to regulate signaling through these pathways. Whether the lipid-bound open conformer impacts the nuclear functions of neurofibromin 2^11,46^ remains to be determined.

## Methods

### DNA constructs and protein preparation

The neurofibromin 2 constructs, residues 1–312 and 1–339, were cloned into pGEX-2T expression vector (GE Life Sciences) using the full-length human neurofibromin 2 plasmid (addgene plasmid number 11629, Supplementary Table 4). The lipid binding deficient mutant (T59V, W60E, R309Q, R310Q) was generated by site-directed mutagenesis from the wild-type neurofibromin 2 FERM domain by using the QuickChange site-directed mutagenesis kit (Agilent Technologies). For functional studies in mammalian cells, wild type and mutant human full-length neurofibromin 2 were cloned using restriction-free cloning method^47^ into a modified pCDNA3 vector (Invitrogen) containing an *N*-terminal flag tag. All plasmid constructs were sequenced and verified.

We cloned the full-length neurofibromin 2 lipid binding deficient and A585W-R588K (AR) mutants into the pGEX-6P-1 plasmid using the restriction-free cloning method and site-directed mutagenesis while we cloned the patient-derived W60C mutant into the PGEX vector.

The neurofibromin 2 proteins (residues 1–339 and 1–595) were expressed in *Escherichia coli* strain BL21(DE3) Rosetta2 (Novagen) at 25 °C for 20 h and cells were induced for protein expression using either 0.05 mM or 0.2 mM IPTG. These cells were harvested by centrifugation at 5,000× *g* for 15 min and lysed by sonication in lysis buffer (20 mM Tris, pH 7.5, 400 mM NaCl, and 0.1 mM EDTA) and the lysate was clarified at 100,000× *g* for 30 min. The supernatant was loaded onto a GST column equilibrated with lysis buffer and the proteins were eluted with 10 mM glutathione in lysis buffer. The proteins were dialyzed overnight in 20 mM Tris (pH 7.5), 400 mM NaCl, 2 mM DTT, and 0.1 mM EDTA and digested with PreScission protease at 4 °C and these proteins were further purified by size exclusion chromatography (SEC) using a 26/60 Superdex 75 column (GE Healthcare) that was pre-equilibrated with 20 mM Tris (pH 7.5), 400 mM NaCl, 1 mM DTT, and 0.1 mM EDTA. For HDX studies, the human full-length GST-tagged neurofibromin 2 supernatant was loaded onto a GST column equilibrated with lysis buffer and the proteins were eluted with 10 mM glutathione in lysis buffer. The proteins were dialyzed overnight in 20 mM Tris (pH 7.5), 500 mM NaCl, 2 mM DTT, 2% glycerol, and 0.1 mM EDTA, and digested with PreScission protease at 4 °C. Proteins were further purified by SEC using a 16/60 Superdex 200 column (GE Healthcare) that was pre-equilibrated with 20 mM Tris (pH 7.5), 500 mM NaCl, 1 mM DTT, 2% glycerol, and 0.1 mM EDTA^48^. All the proteins were concentrated, and aliquots were frozen in liquid N_2_ and stored at −80 °C.

### Neurofibromin 2/PIP_2_ co-crystallization

Our initial attempts to obtain crystals of the neurofibromin 2 FERM domain (residues 1–312) in complex with PIP_2_ were unsuccessful. We used varying concentrations of PIP_2_diC_8_ ranging from 20 to 500 μM, but were unable to obtain crystals. Thus, we used a larger neurofibromin 2 construct (residues 1–339) and 100 μM lipid and screened several commercially available crystallization screens (over 1,000 conditions) and obtained one single crystallization hit from the Index screen of Hampton Research within 1 day only at 20 °C. We improved these crystals by incubating 300 μM neurofibromin 2 with 300 μM PIP_2_diC_8_ (Avanti Polar Lipids) on ice for 12 h and by removing the precipitate by centrifugation (15,800× *g* for 30 min at 4 °C). Plate-like crystals grew within 2 days by hanging drop vapor diffusion (1 μl sample plus 1 μl reservoir) from 100 mM HEPES (pH 7.5) and 50 mM MgCl_2_(H_2_O)_6_ and by varying polyethylene glycol (PEG 550 monomethyl ether) concentrations from 25% to 35% at 20 °C that diffracted X-rays at beamline 22ID at the Advanced Photon Source, Argonne National Laboratory to ∼3 Å Bragg spacings. Crystals were directly flash frozen and stored in liquid nitrogen at 100 K.

### X-ray data collection and processing

X-ray diffraction data were collected on beamline 22ID at the Advanced Photon Source at Argonne National Laboratory and integrated and scaled using XDS and AIMLESS as implemented in autoPROC^49^. Isotropic (to 3.09 Å) and anisotropic (to 2.61 Å) data reduction statistics are provided in Supplementary Table 1.

### Structure determination and crystallographic refinement

Phases were obtained by molecular replacement using the program MOLREP^50^ by using the neurofibromin 2 FERM domain structure (PDB)^34^ as the search model. Molecular replacement solutions were only obtained when we truncated the model to residues 20–309. Two molecules in the asymmetric unit were identified in space group *P*2_1_ resulting in a calculated volume to mass ratio of 2.68 Å^3^/Da, corresponding to a solvent content of 0.5455.

For crystallographic refinement, we used anisotropically scaled high-resolution data to 2.61 Å with an ellipsoidal completeness of 0.903. The neurofibromin 2/PIP_2_ complex structure was refined by performing maximum likelihood as implemented in BUSTER^51^ by imposing target restraints. The model was improved by non-crystallographic symmetry restraints through local structure similarity restraints^52^. Iterative cycles of model building were performed using Coot^53^, and model bias was minimized by building into composite omit maps. The model was further refined and improved by using TLS groups for each polypeptide chain with BUSTER. The missing extended α-helix (residues 310–339) was unambiguously resolved with clear electron density after initial refinement with BUSTER and was built manually using α-helical restraints in Coot. Optimized PIP_2_ ligand coordinates and ligand restraints were obtained from the Grade web server (grade.globalphasing.org). The electron density maps were sharpened using Coot to ensure the directionality and identity of the α-helices in particular at this moderate resolution. The final model contains two polypeptide chains, two phosphates, and two PIP_2_ molecules in the asymmetric unit. The quality of the final model was assessed using MolProbity^54^, which revealed no outliers with >98% of the amino acid residues in the favored region of the Ramachandran plot. Crystallographic refinement statistics are provided in Supplementary Table 2.

### Lipid co-sedimentation assay

Lipid binding to wild type and mutant neurofibromin 2 FERM domains was assayed as described previously^55^. Briefly, lipid vesicles of phosphatidylcholine (PC) and PIP_2_ were prepared in chloroform to a final composition of 80% chicken egg PC and 20% porcine brain PIP_2_ (Avanti polar lipids). The lipid mixture was dried under Argon stream and resuspended in 20 mM Tris (pH 7.5), 400 mM NaCl, 0.1 mM EDTA, and 2 mM DTT (resuspension buffer). Small unilamellar vesicles were then produced by sonication after incubating at 37 °C for 60 min. Samples containing 50 μg of total lipid in 15 μl suspension and 50 μM wild type or mutant neurofibromin 2 domain proteins were incubated at 4 °C for 1 h followed by centrifugation at 100,000× *g* for 30 min. The supernatant and pellet were separated carefully and the pellet was washed twice and resuspended in 15 μl resuspension buffer. The supernatant and pellet samples were analyzed by SDS-PAGE.

### Differential HDX-Mass Spectrometry

HDX-MS analysis of the target protein involves two stages, first peptide identification and second on-exchange HDX. For peptide identification, peptic peptides were fragmented using data-dependent MS/MS selecting top five most abundant ions per scan event using an Orbitrap mass spectrometer (Q Exactive, ThermoFisher) coupled to an automated liquid handling system and HPLC^56^. Peptide identification was performed by submitting raw MS/MS data files to Mascot (Matrix Science). Peptides with a MASCOT score greater than 20 were included in the HDX peptide set and the search was repeated against a decoy (reverse) sequence. Ambiguous identifications were not included in the HDX peptide set. MS/MS spectra for these peptides were verified by manual inspection.

The following procedure was used to perform on-exchange HDX. Neurofibromin 2 (10 μM) was incubated with the pure PIP_2_ micelles at a 1:10 protein-to-ligand molar ratio for 1 h at room temperature. Next, 5 μl of sample was diluted into 20 μl D_2_O buffer (20 mM Tris-HCl, pH 7.4, 150 mM NaCl, 2 mM DTT) and incubated for various time points (0, 10, 60, 300, and 900 s) at RT. The deuterium exchange was then slowed by mixing with 25 μl of ice cold 3 M urea and 1% trifluoroacetic acid. Quenched samples were immediately frozen and remained on dry ice until they were injected into the HDX platform. Upon injection, samples were passed through an immobilized pepsin column (2 mm × 2 cm) at 200 μl min^−1^ and the digested peptides were captured on a 2 mm × 1 cm C_8_ trap column (Agilent) and desalted. Peptides were separated using a 2.1 mm × 5 cm C_18_ column (1.9 μl Hyperkin Gold, Thermo Scientific) with a 5 min linear gradient of 4–40% CH_3_CN and 0.3% formic acid. Both protein pepsin digestion and peptide separation were conducted at 4 °C to aid in deuterium retention. MS data were acquired using on the Orbitrap described above using a measured resolving power of 65,000 at *m*/*z* 400. All on-exchange HDX analyses were performed in triplicate, with single preparations of each protein/ligand complex. The intensity weighted mean *m*/*z* centroid value of each peptide envelope was calculated using HDX Workbench^57^ and these values were converted into percentage of deuterium incorporation. This was accomplished by determining the observed averages of the undeuterated and fully deuterated spectra and using the conventional formula described elsewhere^58^. Statistical significance for the differential HDX data is determined by an unpaired *t*-test for each time point^57^. Back-exchange corrections were made on the basis of an estimated 70% deuterium recovery and accounting for the known 80% deuterium content of the deuterium exchange buffer.

The HDX data from all overlapping peptides were consolidated to individual amino acid values using a residue averaging approach where for each residue, the deuterium incorporation values and peptide lengths from all overlapping peptides were assembled. A weighting function was applied in which shorter peptides were weighted more heavily than longer peptides. Each of the weighted deuterium incorporation values were then averaged to produce a single value per each amino acid. The initial two residues of each peptide, as well as prolines, were omitted from the calculations^59^.

### MST Assay

Specific binding of PIP_2_diC8 and LATS1 (residues 69 to 100) to *N* terminally GST-tagged full-length neurofibromin 2 proteins (wild type, AR, and lipid binding deficient) were measured by the MST method. These proteins were either labeled with maleimide conjugated cysteine reactive or NHS conjugated lysine reactive NT-647 red dyes. Malemide labeling dyes were used for PIP_2_ titrations to avoid interference with PIP_2_ binding site. Unlabeled PIP_2_diC8 (10 mM) or LAST1 residues 69–100 (2 mM) was titrated into a fixed concentration of labeled proteins (5 μM). Binding reactions were carried out in a buffer containing 20 mM Tris (pH 7.5), 300 mM NaCl, and 0.01% Tween-20. Before loading the samples, the reactions were incubated for 10 minutes. Samples were loaded into NT.115 standard treated capillaries (Nanotemper Technologies) and data were measured using Monolith NT.115 pico apparatus (Nanotemper Technologies). The data were recorded at room temperature using the red LED at 20% (GREEN filter; excitation 515–525 nm, emission 560–585 nm) and IR-Laser power at either 20% or 40%. Data analyses were performed with NTAnalysis and only the data with same IR-Laser power were averaged and were plotted using “logistic” function in the Origin software.

### Cell culture and transfection conditions

HEK293 cells were purchased from ATCC, hSC2λ cells were a gift from Dr. Margaret Wallace (https://www.nature.com/articles/labinvest201688), and SC4 cells were a gift from Dr. Helen Morrison^7^. The cell lines used in this study were authenticated by short tandem repeat DNA profiling (DDC Medical). In addition, the cells were tested every 3 months for mycoplasma contamination. HEK293, hSC2λ, and SC4 cells were maintained in low glucose Dulbecco’s Modified Eagle’s Medium (Gibco) + 10% fetal bovine serum (Atlas Biologicals) and antibiotics (100 units ml^−1^ penicillin and 100 μg ml^−1^ Streptomycin) (Gibco) at 37 °C in a humidified atmosphere of 5% CO_2_ (v/v). Experiments were executed with cells grown to 70–80% confluence. Transfections were done using Lipofectamine 2000 (Invitrogen, Carlsbad, CA), as previously described^60^.

### Immunoprecipitation and Rac1-GTP pull-down

Cells were transfected with 8 μg of plasmid DNA (empty vector control or different neurofibromin 2 alleles) and Rac1-GTP levels were determined using a pull-down approach employing a fusion protein comprised of the p21-binding domain of PAK1 fused to GST, according to the manufacturer’s instructions (Millipore, #17-441).

### BrdU Incorporation Assay

Cells were seeded at of 2 × 10^5^ cells/ well in 6-well dishes. On the following day, cells were transfected with 2 μg of plasmid DNA (empty vector control or different neurofibromin 2 alleles). After 24 h of transfection, the cells were trypsinized and were seeded at 2 × 10^4^ cells/well 96-well tissue culture dishes. At 48 h, 72 h, and 96 h post transfection, the cells were processed and analyzed using a BrdU cell proliferation assay kit following manufacturer’s instructions (Millipore, #2750). Plates were read with a spectrophotometer microplate reader (450 nm) (SpectraMax M5; Molecular Devices).

### Data availability

Coordinates and structure factors have been deposited in the Protein Data Bank under accession code 6cds. Other data are available from the corresponding author upon request.

## Electronic supplementary material

Supplementary Information (PDF 41078 kb)

Description of Additional Supplementary Files(PDF 169 kb)

Supplementary Data 1(XLSX 61 kb)
