# Supplementary material for: Lipid binding promotes the open conformation and tumor-suppressive activity of neurofibromin 2
Source: Nat Commun. 2018 Apr 6;9:1338. doi: 10.1038/s41467-018-03648-4 (PMC5889391; doi:10.1038/s41467-018-03648-4)
Supplement: Supplementary file 1 — Supplementary Information (PDF 41078 kb) [file 41467_2018_3648_MOESM1_ESM.pdf]

Supplementary Information

Lipid binding promotes the open conformation and tumor-suppressive activity of neurofibromin 2

Chinthalapudi *et al.*

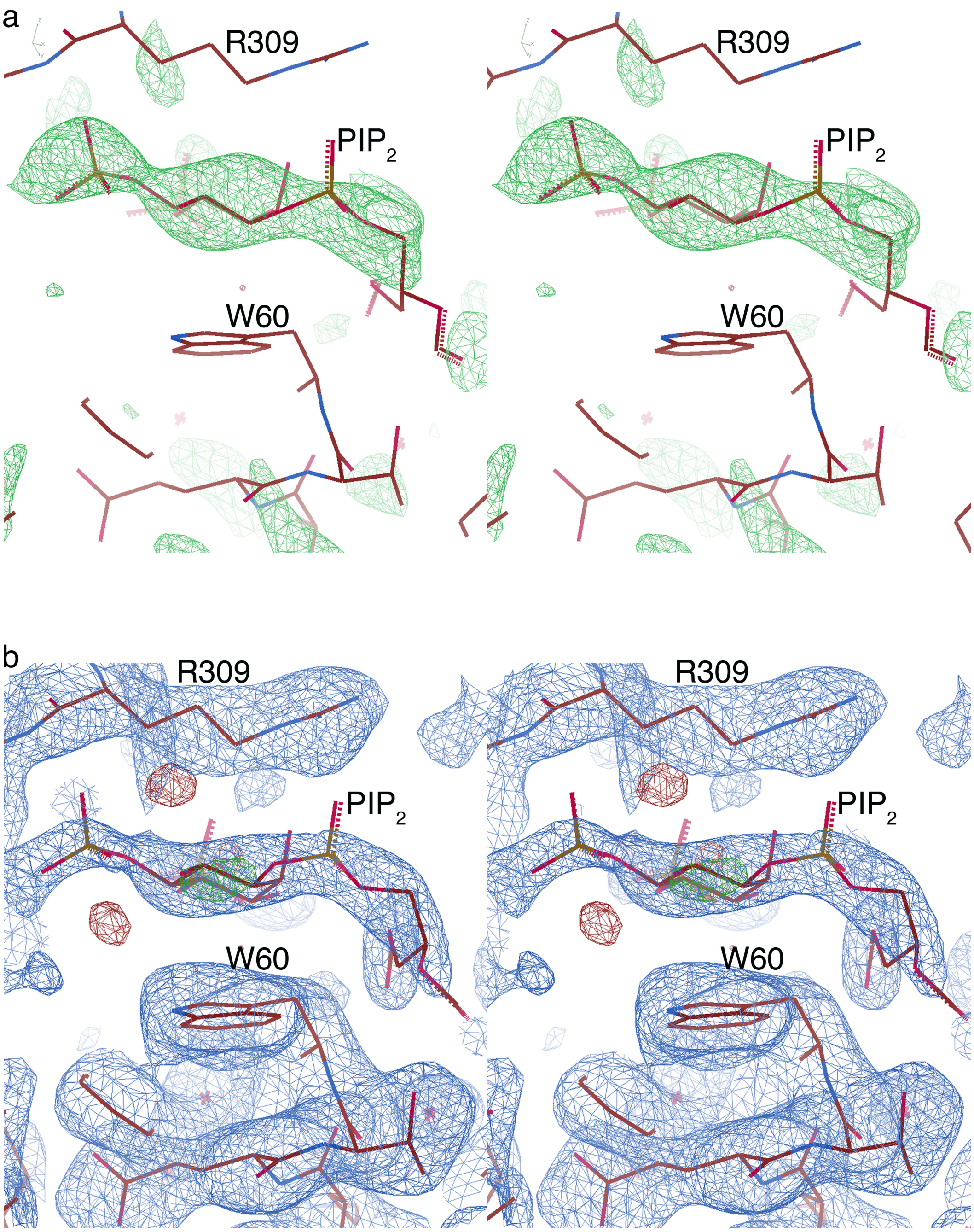

Supplementary Figure 1
PIP_2 binding to chain "A" of neurofibromin 2

(a) Cross-eyed stereo view of the final *F*o - *F*c omit electron density maps (in green) contoured at 2.5 σ of chain "A" of the neurofibromin 2/PIP_2 complex at 2.61 Å resolution. Key neurofibromin 2 binding residues, W60 and R309, are indicated.

(b) Cross-eyed stereo view of the final 2*F*o - *F*c (blue) and *F*o - *F*c (green) electron density maps contoured at 0.85 σ (blue) and 2.8 σ (green and red for positive and negative electron density, respectively).

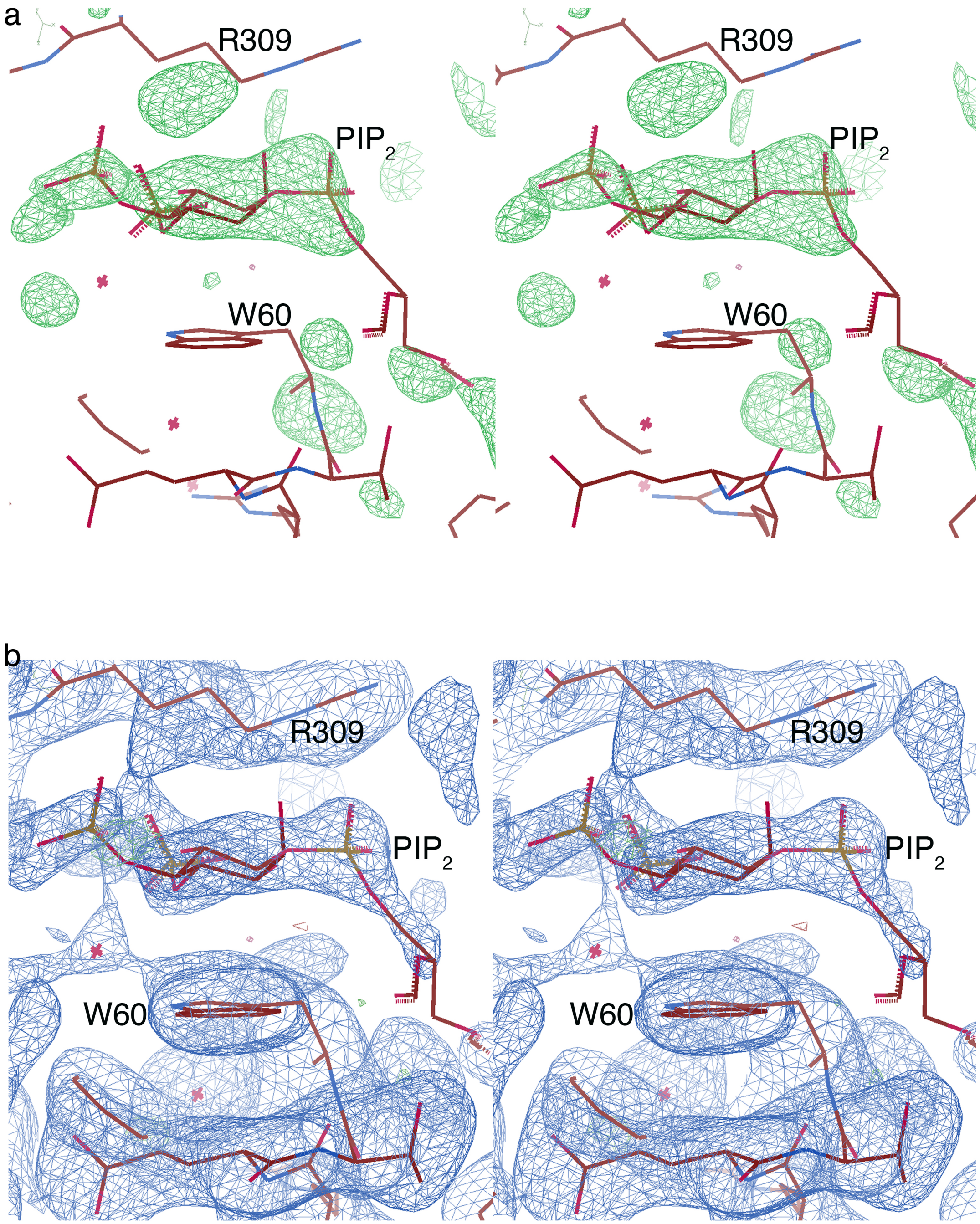

Supplementary Figure 2

PIP_2 binding to chain “B” of neurofibromin 2

(a) Cross-eyed stereo view of the final *F*o - *F*c omit electron density maps (in green) contoured at 2.5 σ of chain “B” of the neurofibromin 2/PIP_2 complex at 2.61 Å resolution.

(b) Cross-eyed stereo view of the final 2*F*o - *F*c (blue) and *F*o - *F*c (green) electron density maps contoured at 0.75 σ (blue) and 3 σ (green).

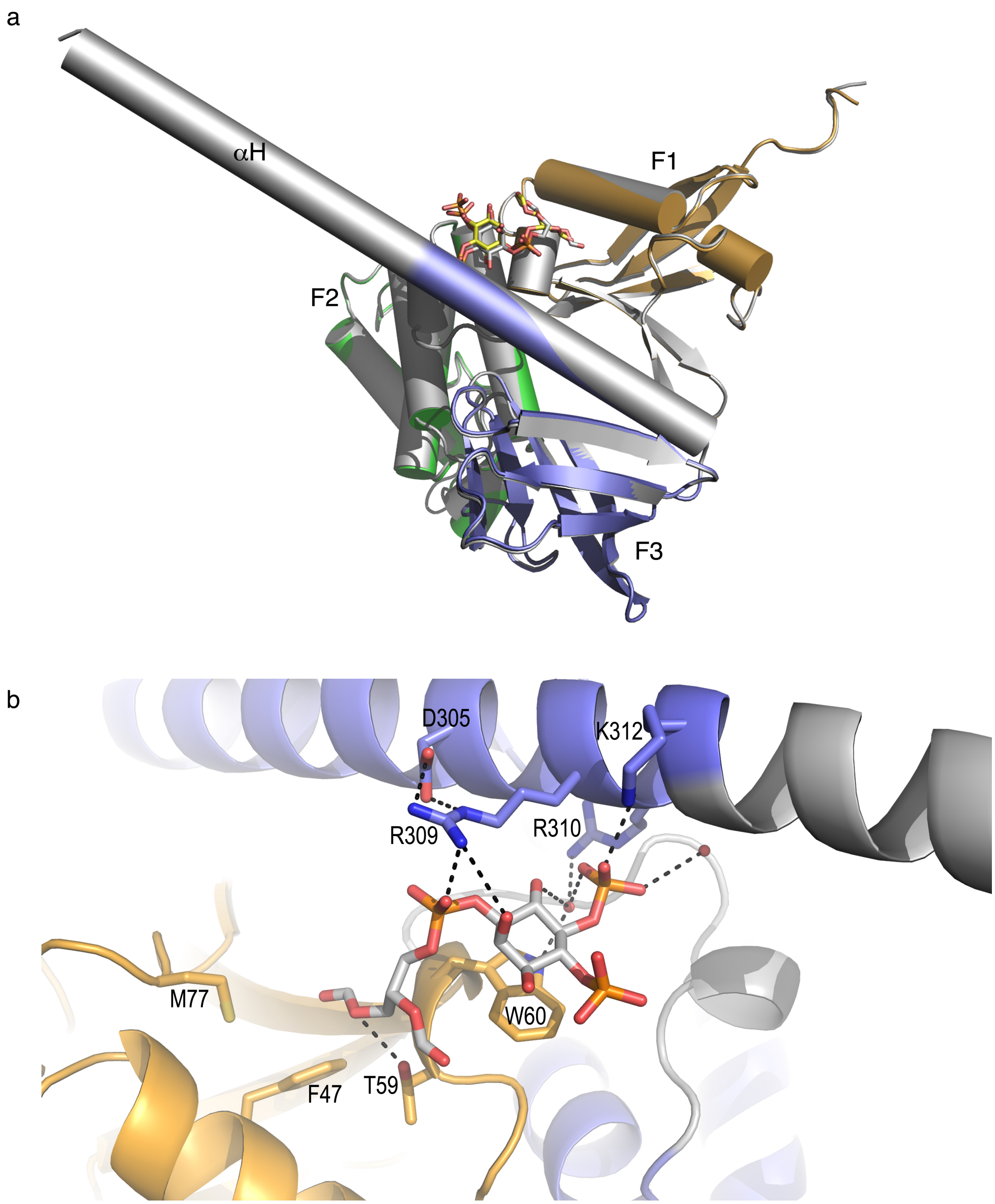

Supplementary Figure 3

(a) Superposition of the two lipid-bound neurofibromin 2 polypeptide chains in the asymmetric unit, shown as a gray (chain “B”) and colored (chain “A”) cartoon (F1, residues 18-98, orange; F2, 111-213, green; F3, 221-312, blue), respectively. The latter (chain “A”) with its bound PIP_2 (shown in sticks) carbons in yellow. The α-helix from the central helical domain, αH, is shown in gray (residues 315-339). Residues 15-339 of one subunit can be superimposed onto the second subunit in the asymmetric unit with *r.m.s.d.* of 0.278 Å for 2,100 atoms.

(b) Close-up view of the PIP_2 binding site in the second molecule in the asymmetric unit. The carbon atoms of PIP_2 are shown in gray, of the neurofibromin 2 F1 subdomain in light orange, and F3 in blue. Hydrogen bonds are indicated and key binding residues are labeled.

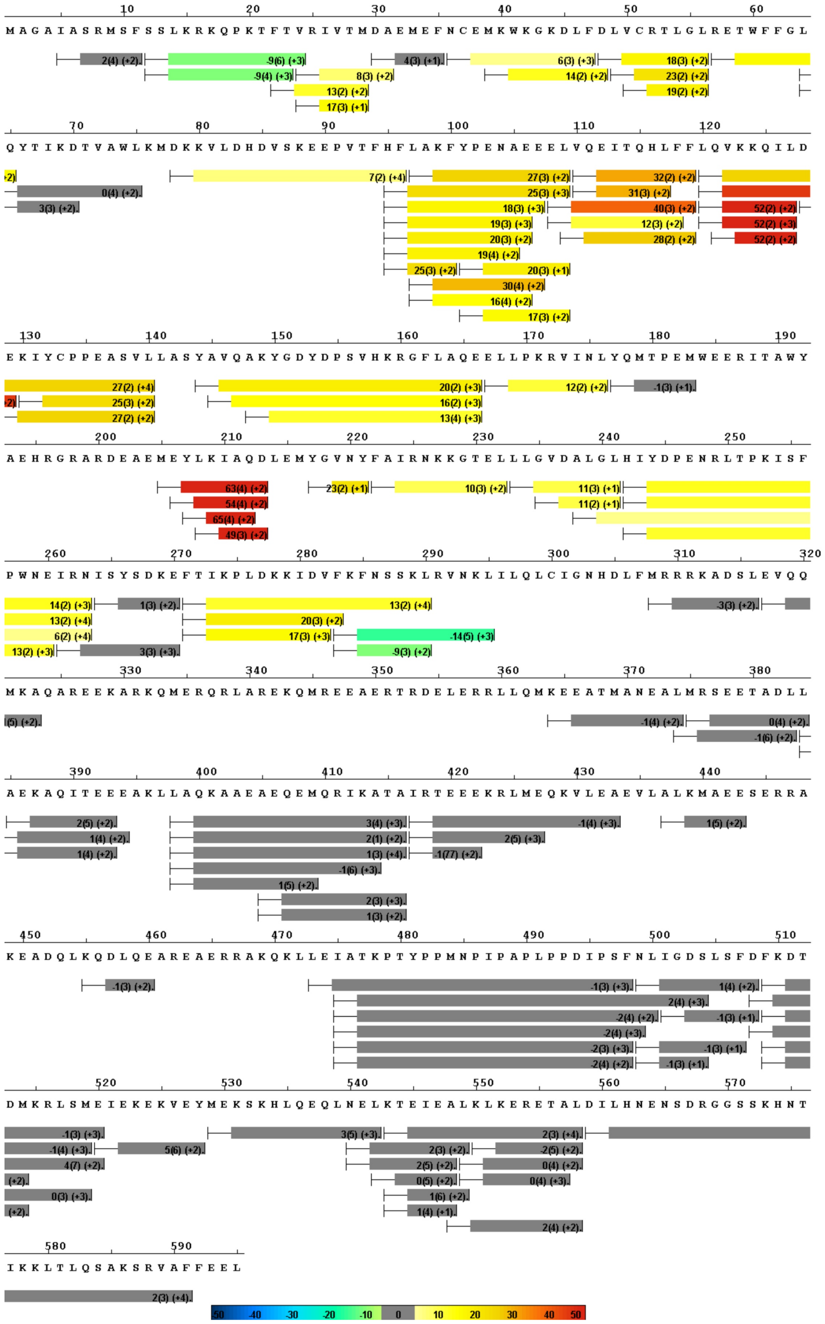

Supplementary Figure 4

Differential HDX mapping of full-length neurofibromin 2 upon binding to PIP_2 micelles

HDX single amino acid consolidation view of full-length neurofibromin 2 upon binding to PIP_2 micelles. Percentages of deuterium differences are color-coded according to the smooth color gradient key. A negative value represents decreased deuterium incorporation or stabilization while a positive value represents increased deuterium incorporation or destabilization in the corresponding region of the protein. Gray, no statistically significant changes between compared conditions; blank, regions that have no sequence coverage or include prolines that have no amide hydrogen exchange activity. The deuterium incorporation data from all overlapping peptides were consolidated to individual amino acid values using a residue averaging approach[1].

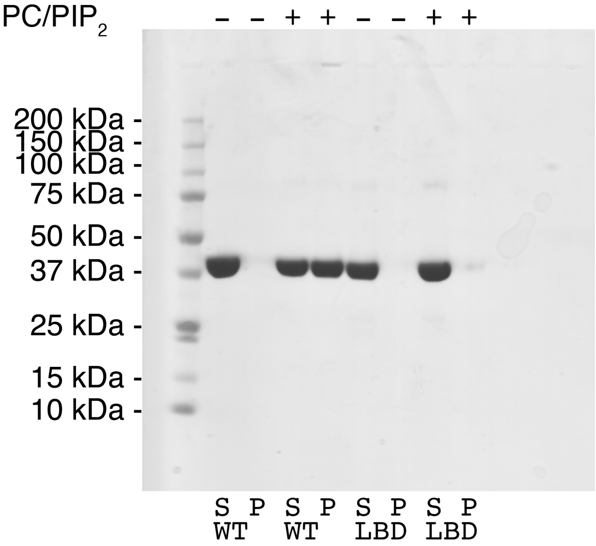

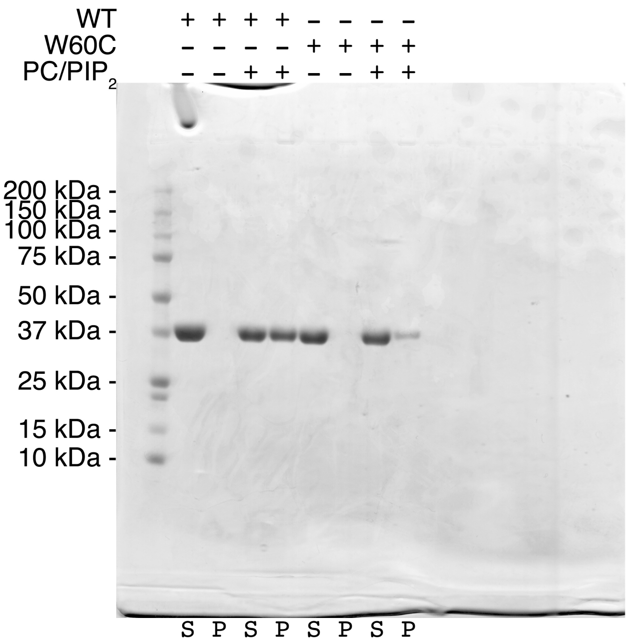

Supplementary Figure 5

Full images of Figure 3 (a, b)

The conformation of neurofibromin 2 dictates its binding

(left) Lipid co-sedimentation analysis of the PIP_2 binding to wild type and our lipid binding deficient mutant neurofibromin 2. Wild type neurofibromin 2 (residues 1-339) is soluble in the absence of PIP_2 and pellets in the presence of PIP_2. Mutant (T59V, W60E, R309Q, R310Q) neurofibromin 2 (residues 1-339) remains soluble in the absence and presence of PIP_2. S, supernatant; P, pellet; WT, wild type; LBD, lipid binding deficient mutant (T59V, W60E, R309Q, R310Q).

(right) Lipid co-sedimentation analysis of the PIP_2 binding to wild type and disease derived mutant neurofibromin 2. Wild type neurofibromin 2 (residues 1-339) is soluble in the absence of PIP_2 and pellets in the presence of PIP_2. Mutant (W60C) neurofibromin 2 (residues 1-339) is soluble in the absence of PIP_2 while a small fraction pellets in the presence of PIP_2. S, supernatant; P, pellet; WT, wild type.

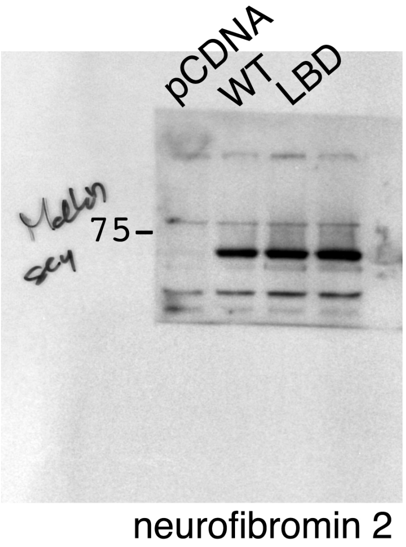

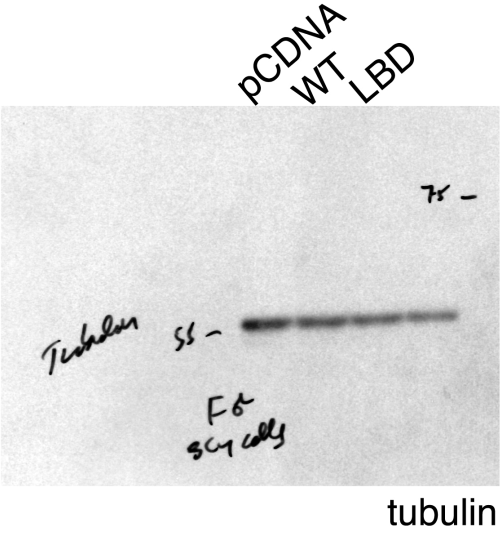

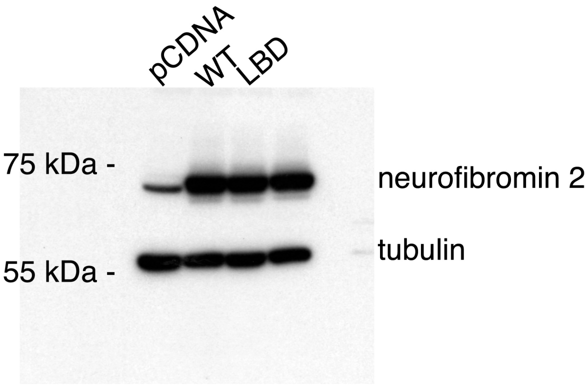

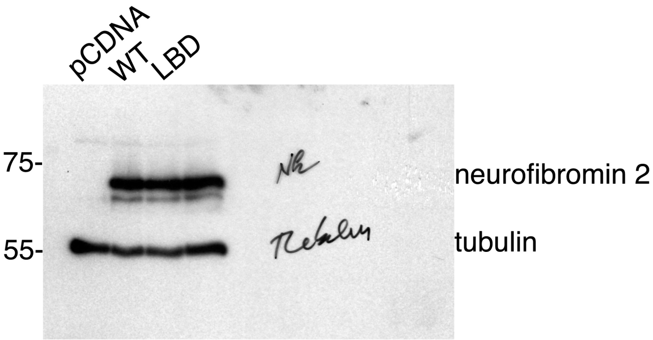

Supplementary Figure 6

Full images of Figure 4 (a)-(c)

Lipid binding deficient mutants of neurofibromin 2 display impaired inhibition of cell proliferation

(top) SC4, **(left bottom)** HEK293T, or **(right bottom)** hSCλ-shNF2 cells were transfected with expression vectors for wild type and lipid binding deficient neurofibromin 2 or empty vector control (pCDNA). Total cell numbers were counted over 72 hours. Means of each data point were calculated from three independent biological replicates conducted in triplicate. Error bars represent ±S.D. Immunoblot analysis was used to verify similar expression levels of the indicated neurofibromin 2 alleles. Tubulin was used as a control. The blots shown are representative of three biological replicates. Unlabeled fourth lanes are samples of a different line of investigation, not part of this study.

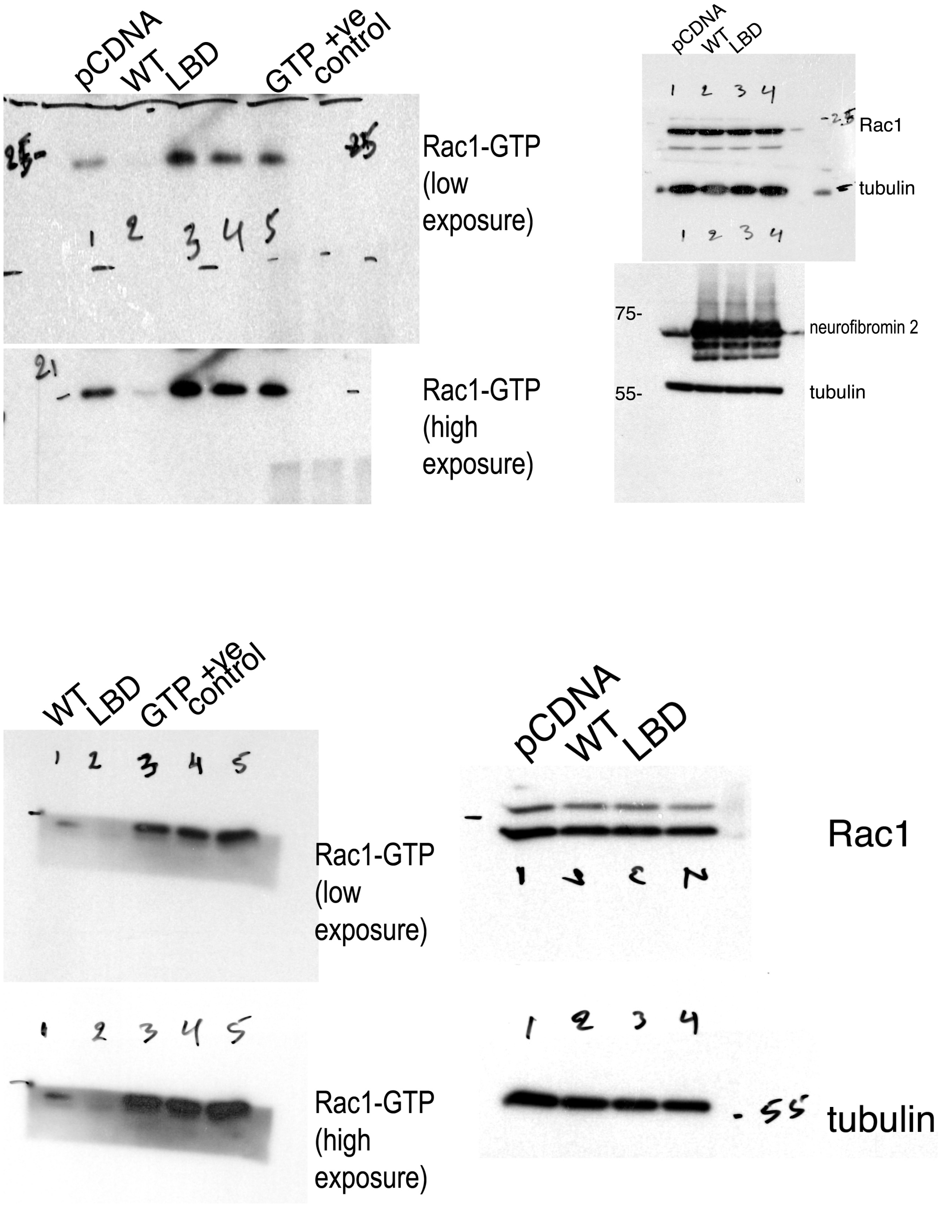

Supplementary Figure 7

Full images of Figure 5 (a, b)

The lipid binding deficient mutant of neurofibromin 2 displays impaired inhibition of Rac1 activation and YAP activity

(top) 293T or **(bottom)** SC4 cells were transfected with expression vectors for wild type or lipid binding deficient neurofibromin 2 or empty vector control (pCDNA) and levels of active Rac1 (Rac1-GTP) were assessed after 48 hours. Levels of total Rac1, neurofibromin 2, and tubulin were assessed as controls. The blots shown are representative of three biological replicates. Unlabeled lanes are samples of a different line of investigation, not part of this study.

Supplementary Table 1
X-ray data reduction statistics for human neurofibromin 2 (residues 1 to 339) in complex with PIP_2

Isotropic Scaling

Space group	$P\,2_1$
Unit cell dimensions	
a, b, c	42.31 Å, 96.59 Å, 106.45 Å
α, β, γ	90°, 99.33°, 90°
Resolution, overall	48.29 Å - 3.09 Å
Last shell	3.15 Å - 3.09 Å
Total measurements	53,346
Nr. of unique reflections, overall	15,264
Last shell	2,769
Wavelength	1.0 Å
$R_{p.i.m,.}$ overall	0.074
Last shell	0.333
I/σ(I), overall	9.5
Last shell	2.2
Completeness, overall	0.985
Last shell	1.000
Multiplicity, overall	3.5
Last shell	3.6
$CC_{1/2}$, overall	0.993
Last shell	0.826

Anisotropic Scaling

Space group	$P\,2_1$
Unit cell dimensions	
a, b, c	42.31 Å, 96.59 Å, 106.45 Å
α, β, γ	90°, 99.33°, 90°
Resolution, overall	48.29 Å – 2.62 Å
Last shell	2.81 Å - 2.62 Å
Total measurements	66,489
Nr. of unique reflections, overall	19,196
Last shell	1,274
Wavelength	1.0 Å
$R_{p.i.m,.}$ overall	0.091
Last shell	0.688
I/σ(I), overall	7.9
Last shell	0.9
Completeness (spherical), overall	0.754
Last shell	0.115
Completeness (ellipsoidal), overall	0.903
Last shell	0.396
Multiplicity, overall	3.5
Last shell	2.3
$CC_{1/2}$, overall	0.991
Last shell	0.338

Supplementary Table 2
Crystallographic refinement statistics for human neurofibromin 2 (residues 1 to 339) in complex with PIP_2

The final model contains two polypeptide chains, two phosphates, and two PIP_2 molecules in the asymmetric unit.

Resolution, overall	48.30 Å – 2.62 Å
Last shell	2.76 Å – 2.62 Å
No. of reflections, working	18,234
Test set	962
R-factor, overall	0.188
Last shell	0.274
R-free, overall	0.237
Last shell	0.404
No. of non-hydrogen atoms:	
Protein	5,395
Ligands	96
Solvent	222
Average B-factor:	
Protein	42.53 $Å^2$
Ligands	108.36 $Å^2$
Solvent	30.09 $Å^2$
R.m.s.d. from ideal values:	
Bond lengths	0.010 Å
Bond angles	1.06°
Ramachandran favored	98.14%
Ramachandran allowed	1.86%
Ramachandran outliers	0

Supplementary Table 3
Neurofibromin 2 residues interacting with PIP_2 as seen in the neurofibromin 2/PIP_2 crystal structure

(a) Hydrophobic interactions

FERM residues (chain A)	PIP_2 atoms (chain A)	Distance [Å]
Arg-309 CD	C6	4.10
Arg-309 CB	C6	4.55
Trp-60 CD2	C1	3.45
Trp-60 CE3	C1	3.61
Trp-60 CE3	C2	3.91
Trp-60 CE3	C3C	3.90
Trp-60 CZ3	C3	3.74

FERM residues (chain B)	PIP_2 atoms (chain B)	Distance [Å]
Arg-309 CD	C6	3.87
Arg-309 CB	C6	4.37
Trp-60 CD2	C1	3.74
Trp-60 CD2	C3	3.98
Trp-60 CE3	C3	3.66
Trp-60 CE2	C5	3.60
Trp-60 CG	C1	3.76
Trp-60 CH2	C1	3.85
Trp-60 CZ3	C3	3.59
Trp-60 CZ2	C5	3.75
Leu-306 CD2	C1	4.23

(b) Hydrophilic interactions

FERM residues (chain A)	PIP_2 atoms (chain A)	Distance [Å]
Arg-309 NH1	O12	2.29
Arg-309 NH1	O2	3.60
Arg-309 NH2	O12	3.32
Arg-309 NE	O12	2.92
Arg-309 O	O51	2.78
Arg-310 N	O51	3.19
Thr-59 OG1	O2C	3.82
Thr-59 OG1	O1A	3.90

FERM residues (chain B)	PIP_2 atoms (chain B)	Distance [Å]
Arg-309 NH1	O11	2.85
Arg-309 NH1	O2	3.55
Arg-309 NH2	O11	3.89
Arg-309 NE	O1	3.87
Arg-309 O	O51	3.37
Arg-310 N	O51	3.95
Thr-59 OG1	O3C	3.08
Thr-59 OG1	O1A	3.71
Arg-57 O	O1A	3.85
Leu-306 O	O53	3.70

Supplementary Table 4
Primer sequences

Construct pGEX6P1 vector specific
Forward primer (5' →3') TGACTGACGATCTGCCTCG
Reverse primer (5' →3') GGGCCCCTGGAACAGAAC

Construct hNF2-FERM_1-339 gene specific
Forward primer (5' →3') CTGTTCCAGGGGCCCATGGCCGGGGCCATCGCTTC
Reverse primer (5' →3') GCAGATCGTCAGTCAGAGGCGCTGCCGCTCCATC

Construct pCDNA3 vector specific
Forward primer (5' →3') CAAATCAAAGAGGTCCTTCCCTTTCC
Reverse primer (5' →3') CCAGGGAGGAGAAGGCTAGAAAG

Construct hNF2_1-595 gene specific
Forward primer (5' →3') GGAAAGGGAAGGACCTCTTTGATTTG
Reverse primer (5' →3') CTTTCTAGCCTTCTCCTCCCTGG

Construct pET32a vector specific
Forward primer (5' →3') GGTATGAAAGAAACCGCTGCTG
Reverse primer (5' →3')
GGGCCCCTGGAACAGAACTTCCAGACCAGAAGAATGATGATGATG

Construct GATG hNF2_1-595 gene specific
Forward primer (5' →3')
CTGGAAGTTCTGTTCCAGGGGCCCATGGCCGGGGCCATCGCTTC
Reverse primer (5' →3')
CAGCAGCGGTTTCTTTCATACCCTAGAGCTCTTCAAAGAAGGCC

Construct hNF2_A585W-R586K mutant
Forward primer (5' →3')
TAAAAAGCTCACCTTGCAGAGCTGGAAGTCCAAAGTGGCCTTCTTTGAAGAGCTC
Reverse primer (5' →3')
GAGCTCTTCAAAGAAGGCCACTTTGGACTTCCAGCTCTGCAAGGTGAGCTTTTTA

Construct hNF2_T59V-W60E mutant
Forward primer (5' →3')
GGACTCTGGGGCTCCGAGAAGTCGAGTTCTTTGGACTGCAGTACA
Reverse primer (5' →3')
TGTACTGCAGTCCAAAGAACTCGACTTCTCGGAGCCCCAGAGTCC

Construct hNF2_R309Q-R310Q mutant
Forward primer (5' →3')
TCCAGCTATGTATCGGGAACCATGATCTATTTATGCAGCAGAGGAAAGCCGATTCTTTGGAAG
Reverse primer (5' →3')
CTTCCAAAGAATCGGCTTTCCTCTGCTGCATAAATAGATCATGGTTCCCGATACATAGCTGGA

Construct hNF2-W60C mutant
Forward primer (5' →3') GCTCCGAGAAACCTGCTTCTTTGGACTGCAG
Reverse primer (5' →3') CTGCAGTCCAAAGAAGCAGGTTTCTCGGAGC

Supplementary References

1. Keppel TR, Weis DD. Mapping residual structure in intrinsically disordered proteins at residue resolution using millisecond hydrogen/deuterium exchange and residue averaging. *J Am Soc Mass Spectrom* **26**, 547-554 (2015).
