# Supplementary material for: Lipid binding promotes the open conformation and tumor-suppressive activity of neurofibromin 2
Source: Nat Commun. 2018 Apr 6;9:1338. doi: 10.1038/s41467-018-03648-4 (PMC5889391; doi:10.1038/s41467-018-03648-4)
Supplement: Supplementary file 2 — Description of Additional Supplementary Files(PDF 169 kb) [file 41467_2018_3648_MOESM2_ESM.pdf]

Description of Additional Supplementary Files

File Name: Supplementary Data 1

Description: We provide our HDX dynamics data (absolute deuterium exchange) for the unbound and lipid-bound full-length neurofibromin 2 proteins in the form of all time points of the HDX.
